# A Prediction Model for Sight-Threatening Diabetic Retinopathy Based on Plasma Adipokines among Patients with Mild Diabetic Retinopathy

**DOI:** 10.1155/2023/8831609

**Published:** 2023-10-25

**Authors:** Yaxin An, Bin Cao, Kun Li, Yongsong Xu, Wenying Zhao, Dong Zhao, Jing Ke

**Affiliations:** ^1^Center for Endocrine Metabolism and Immune Diseases, Beijing Luhe Hospital, Capital Medical University, Beijing 101149, China; ^2^Beijing Key Laboratory of Diabetes Research and Care, Beijing 101149, China

## Abstract

**Background:**

Accumulating evidence has suggested a link between adipokines and diabetic retinopathy (DR). This study is aimed at investigating the risk factors for sight-threatening DR (STDR) and establishing a prognostic model for predicting STDR among a high-risk population of patients with type 2 diabetes mellitus (T2DM).

**Methods:**

Plasma concentrations of adipokines were determined by enzyme-linked immunosorbent assay. In the case-control set, principal component analysis (PCA) was performed to select optimal predictive cytokines for STDR, involving severe nonproliferative DR (NPDR) and proliferative DR. Support vector machine (SVM) was used to examine the possible combination of baseline plasma adipokines to discriminate the patients with mild NPDR who will later develop STDR. An individual prospective cohort with a follow-up period of 3 years was used for the external validation.

**Results:**

In both training and testing sets, involving 306 patients with T2DM, median levels of plasma adiponectin (APN), leptin, and fatty acid-binding protein 4 (FABP4) were significantly higher in the STDR group than those in mild NPDR. Except for adipsin, the other three adipokines, FABP4, APN, and leptin, were selected by PCA and integrated into SVM. The accuracy of the multivariate SVM classification model was acceptable in both the training set (AUC = 0.81, sensitivity = 71%, and specificity = 91%) and the testing set (AUC = 0.77, sensitivity = 61%, and specificity = 92%). 110 T2DM patients with mild NPDR, the high-risk population of STDR, were enrolled for external validation. Based on the SVM, the risk of each patient was calculated. More STDR occurred in the high-risk group than in the low-risk group, which were grouped by the median value of APN, FABP4, and leptin, respectively. The model was validated in an individual cohort using SVM with the AUC, sensitivity, and specificity reaching 0.77, 64%, and 91%, respectively.

**Conclusions:**

Adiponectin, leptin, and FABP4 were demonstrated to be associated with the severity of DR and maybe good predictors for STDR, suggesting that adipokines may play an important role in the pathophysiology of DR development.

## 1. Introduction

Diabetic retinopathy (DR), which is a common and specific microvascular complication of type 2 diabetes mellitus (T2DM), remains one of the principal causes of preventable visual disability in adults [1, 2]. With the continuously climbing prevalence of T2DM, the worldwide cases of vision impairment and blindness attributable to DR have increased by 67% and 27%, respectively, from 1990 to 2010 [3]. Research shows that early detection of nonproliferative DR (NPDR) may lead to a 60% reduction in proliferative DR (PDR) and an 83% reduction in blindness [4]. Hence, early recognition, risk identification, and prompt treatment are crucial to delay the progression to sight-threatening DR (STDR), resulting in permanent vision loss and a major burden to society and individuals.

Adipose tissue, initially considered as a reservoir of fat mass, is currently established as an active endocrine organ, functioning in systematic energy and metabolic homeostasis [5]. Adipokines, a range of bioactive molecules derived from adipose tissue, have been demonstrated to be involved in the pathogenesis of T2DM and its complications [6, 7]. Leptin and adiponectin (APN), the most widely studied adipokines, have been suggested as potential biomarkers for DR [8, 9]. In addition, increasing studies have documented that several other adipokines, such as fatty acid-binding protein 4 (FABP4) [10, 11] and omentin-1 [12, 13], are associated with DR and/or its severity. Taken together, it suggests that circulating adipokines might be risk factors for DR and may have the potential to predict the progression of DR.

In the present study, we aimed to investigate the risk factors for STDR and establish a prognostic model for predicting STDR among a high-risk population. Additionally, an individual cohort was used for external validation.

## 2. Methods

All samples were collected with the signed informed consents from all patients, and all related procedures were performed with the approval by the Ethics Committee of the Beijing Luhe Hospital, Capital Medical University.

### 2.1. Study Population

Initially, 132 T2DM with STDR and 281 T2DM with mild NPDR patients were enrolled from the Center for Endocrine Metabolism and Immune Diseases of Beijing Luhe Hospital, Capital Medical University (Beijing, China) between October 2017 and January 2019. Of the 413 subjects, 97 were excluded due to missing data of critical variables, missing stored plasma samples, and the other exclusion criteria listed below. Finally, 106 T2DM patients with STDR (STDR group) and 200 subjects with mild NPDR (mild NPDR group) were enrolled, forming the case-control set. Moreover, 110 T2DM patients with mild NPDR comprised the longitudinal cohort and were followed up for external validation. All patients in the individual cohort underwent two-field fundus photography at least once a year.

The diagnosis of T2DM was made in accordance with the American Diabetes Association (ADA) criteria [14]. Those patients with type 1 diabetes or specific types of diabetes; acute complications of diabetes; fundus lesions owing to an orbital tumor, trauma, malignant hypertension, and a history of any previous intravitreal injection or any other treatment for DR; and acute phase of coronary artery disease or cerebral infarction were excluded.

### 2.2. Diabetic Retinopathy Assessment

All participants received two-field fundus photography, and the photographs were graded independently by an experienced ophthalmologist according to the International Classification of Diabetic Retinopathy scale [15]: (1) no changes in DR, (2) mild nonproliferative diabetic retinopathy (NPDR), (3) moderate NPDR, (4) severe NPDR, and (5) proliferative diabetic retinopathy (PDR). STDR was defined as the combination of severe NPDR and PDR. The severity of retinopathy was determined by the fundus status of the worst affected eye.

### 2.3. Demographic and Laboratory Data Collection

Demographic details of patients were recorded, including age, gender, body mass index (BMI), duration of diabetes, and history of hypertension. BMI was calculated as the weight in kilograms divided by height in meters squared. Blood samples were collected from an antecubital vein for measuring the concentrations of laboratory parameters, including HbA1c, fasting glucose, fasting C-peptide, total cholesterol (TC), triglyceride (TG), serum creatinine (Cr), and urine albumin to creatinine ratio (UACR). Overnight fasting blood samples were collected before 08 : 00 hours. All plasma was produced by standard blood processing; then, aliquots were frozen at −80°C and stored for further analysis, avoiding freeze-thaw cycles.

### 2.4. Enzyme-Linked Immunosorbent Assay (ELISA)

Validation of plasma adipokines, including leptin, APN, FABP4, and adipsin, was performed using ELISA kits following the manufacturer's instructions (Human ELISA kit, Mlbio, China). Intra-assays and interassays for adipsin, leptin, FABP4, and APN gave mean CV values of 10% and 15%, respectively.

### 2.5. Statistical Analysis

The case-control set was randomly divided into training and testing sets according to a ratio of 7 : 3. Using the training cohort, principal component analysis (PCA) was performed to select optimal predictive cytokines for STDR. Support vector machine (SVM) using the “e1071” package was used to examine the possible combination of baseline plasma cytokines to discriminate the mild NPDR participants who will later develop STDR. The SVM in this study adopted a linear kernel (gamma equals to 0.1 and cost (C) equals to 10). The performance of SVM was evaluated by the area under the receiver operating characteristic curve (AUC), sensitivity, and specificity.

Continuous variables were summarized as the median and interquartile range (IQR), and categorical variables were presented in number and percentage (%). Wilcoxon's test was used to compare nonparametric data. Chi-square test was performed for categorical variables. R software version 3.6.3 (R Foundation for Statistical Computing, Vienna, Austria) was used for all analyses. All the statistics were two-sided, with a *p* value less than 0.05 considered to be statistically significant.

## 3. Results

### 3.1. Clinical Characteristics of the Study Participants

The flow chart of this study is illustrated in Supplemental Figure 1. Initially, 132 T2DM with STDR and 281 T2DM with mild NPDR patients were enrolled. After excluding 97 patients who met the exclusion criteria, 306 patients were enrolled, of which 215 subjects were randomly assigned to the training set and 91 cases were into the testing set. In the prospective cohort, 110 T2DM patients with mild NPDR were recruited to validate the performance of the predictive model. The follow-up period of the longitudinal cohort was 36 months. The clinical characteristics of patients are summarized in Table 1. The prevalence of STDR in training and testing sets was 34.9% and 34.1%, respectively.

The median duration of diabetes in the STDR group was longer than in mild NPDR in the testing set (13.17 vs. 6.21 years, *p* = 0.035) rather than that in the training set (10.67 vs. 8.88 years, *p* = 0.282). However, there was no significant difference in age, fasting C-peptide, eGFR, HbA1c, TG, LDL, and TC between the mild NPDR and the STDR group in both training and testing sets (Supplemental Figure 2).

### 3.2. Adipokines Altered in Plasma of STDR

**Figure 1:**
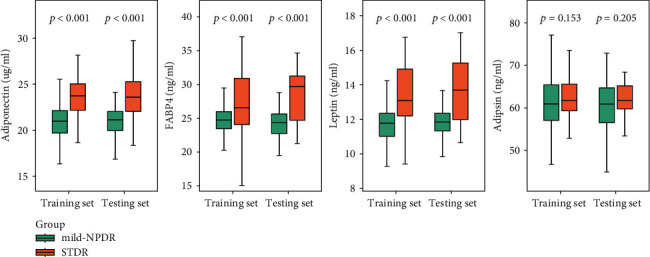
Plasma concentrations of 4 candidate adipokines divided into in mild NPDR group (green color) and STDR group (orange color) in both training and testing sets.

Plasma levels of candidate cytokines were detected using ELISA kits. As shown in Figure 1, median levels of plasma APN, leptin, and FABP4 were significantly higher in the STDR group (23.74 *μ*g/ml, 13.10 ng/ml, and 26.62 ng/ml, respectively) than those in the mild NPDR group (20.98 *μ*g/ml, 11.77 ng/ml, and 24.79 ng/ml, respectively) in the training set. Similarly, plasma concentrations of APN, leptin, and FABP4 were more elevated in the patients with STDR (23.60 *μ*g/ml, 13.69 ng/ml, and 29.77 ng/ml) than in the mild NPDR group (21.13 *μ*g/ml, 11.84 ng/ml, and 24.38 ng/ml, respectively) in the testing set. Concerning adipsin, however, there was no significant diffidence between the STDR and T2DM group in both training and testing sets (61.81 ng/ml vs. 60.91 ng/ml and 61.77 ng/ml vs. 60.90 ng/ml, respectively).

### 3.3. Plasma Adipokine Selection for SVM

In the training set, PCA was performed to compute the relative contribution of each adipokine to the separation of mild NPDR and STDR. The first and second principal components accounted for 74.5% and 16.2% of the variation, respectively. The projection of samples in PCA can be distinguished with relatively small overlapping areas. Adipsin contributed more to the second principal component than the first, while FABP4, APN, and leptin contributed more to the first principal component (Supplemental Figure 3A). The contribution order to the first principal component was FABP4 (28.6%), APN (28.1%), leptin (27.7%), and adipsin (15.6%), respectively (Supplemental Figure 3B). Finally, FABP4, APN, and leptin were integrated into SVM.

### 3.4. Model Training of SVM

**Figure 2:**
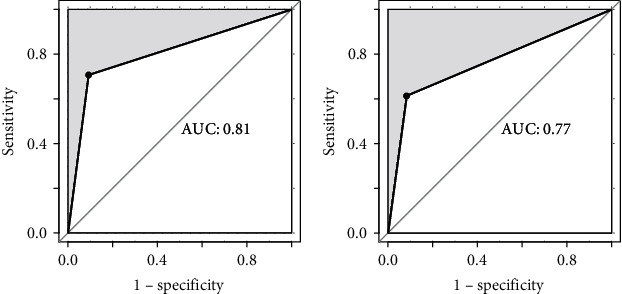
The accuracy of the prediction model indicated by average area under the curve in the training (a) and testing sets (b).

Subsequently, a multivariate SVM classification model was created with a linear kernel using the features obtained from the feature selection process by the PCA. As presented in Figure 2, the accuracy of classification with SVM was acceptable in both the training set (AUC = 0.81, sensitivity = 71%, and specificity = 91%) (Figure 2(a)) and the testing set (AUC = 0.77, sensitivity = 61%, and specificity = 92%) (Figure 2(b)).

### 3.5. External Validation of SVM

**Figure 3:**
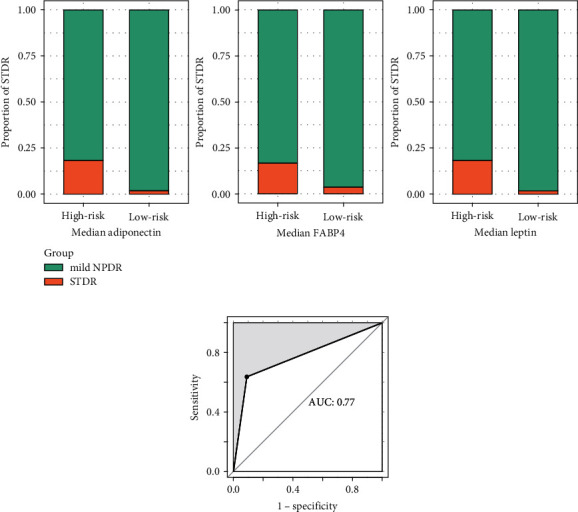
External validation of the prediction model in a prospective cohort. (a) High- and low-risk patients for STDR were grouped by the median value of adiponectin, FABP4, and leptin, respectively. (b) The average area under the curve by SVM in an individual cohort.

110 T2DM mild NPDR patients, the high-risk population of STDR, were enrolled for external validation. Based on the SVM, the risk of each patient was calculated. A total of 11 patients were detected with STDR during the follow-up period. As illustrated in Figure 3(a), high- and low-risk patients were grouped by the median value of APN, FABP4, and leptin, respectively. More patients with STDR occurred in the high-risk group than in the low-risk group among the three predictors. The predictive model was validated by SVM to perform acceptably with the AUC, sensitivity, and specificity of 0.77, 64%, and 91%, respectively (Figure 3(b)).

## 4. Discussion

Due to the considerably higher risk of vision loss and blindness induced by STDR compared to patients with an early degree of DR, it is increasingly urgent and important to discover an effective method to predict the progression to STDR among a high-risk population of DR. In our study, we demonstrated that APN, leptin, and FABP4, confirmed as risk factors for DR, were expected to predict the development of STDR among patients with mild NPDR. In addition, an individual longitudinal cohort with a follow-up period of 3 years was used to external validation of the model, suggesting a comparable and acceptable performance.

In this study, circulating levels of APN, leptin, FABP4, and adipsin were determined in 306 patients with T2DM, displaying a significantly higher level of the three former candidate adipokines in the STDR group than those with mild NPDR. APN, a secreted cytokine primarily derived from adipocytes, exerts a protective role in insulin resistance [16], atherogenesis [17], inflammation [18], and organ fibrosis [19]. In contrast to most of adipokines, the circulating concentrations of APN are reduced in obesity and its complications, including T2DM [20]. High concentrations of APN are associated with a lower risk of T2DM [21]. There is considerable evidence to suggest a link between APN and DR, despite the inclusive results, which were summarized in the meta-analysis and review article [8, 22]. A Mendelian randomization study reported limited evidence for the causal effect of APN on increasing the risk of DR among an Asian population [23]. In animal models, it was demonstrated that APN is primarily located in the retinal vascular endothelium of arterioles [24]. Accompanying the increased vascular permeability, a progressive decrease in retinal APN was detected during the diabetes course [24]. In in vitro experiments using primary human microvascular retinal endothelial cells (HMRECs) under hyperglycemic conditions, APN treatment was shown to ameliorate endothelial barrier dysfunction, as well as decrease inflammatory and oxidative response [25]. Additionally, APN could inhibit angiogenesis of choroidal-retinal endothelial (RF/6A) cells by inhibition of autophagy under high glucose conditions [26].

FABP4, which is a chaperone protein for fatty acids, is widely expressed in the adipocytes, macrophages, and capillary endothelial cells. There is a positive association of FABP4 with obesity [27] and T2DM [28]. The results in our study were consistent with the previous study [29], displaying that FABP4 was positively related to DR severity in patients with T2DM. A 5-year prospective cohort study demonstrated a positive association of baseline FABP4 with DR, and its severity developed in the future [10]. Moreover, FABP4 was detected in the vitreous fluid of PDR patients, showing a higher concentration compared to those in non-PDR patients [11, 30]. A recent study illustrated that inhibition of FABP4 attenuates lipid peroxidation and oxidative stress in both mouse and cell model of DR through regulating PPAR*γ*-mediated ferroptosis, suggesting that FABP4 inhibitor plays a potential in DR treatment [31]. It was reported that in diabetic patients, a reverse association between endothelial dysfunction and FABP4 [32]was demonstrated by inhibiting insulin-signaling pathway or activating the STAT-1 signaling pathway [33, 34]. Additionally, several other possible roles of FABP4 in DR development have been suggested, involving proangiogenic and proliferative [35, 36], as well as proinflammatory properties [37].

Leptin is an adipokine and plays an important role in metabolic homeostasis by inhibiting appetite. Obesity and T2DM are accompanied by elevated levels of leptin and characterized by leptin resistance induced by hyperleptinemia. Our results were in agreement with most of the previous studies, which demonstrated a positive association of serum or vitreous leptin levels with DR or the severity [38–42], while other study failed to show any significant correlation [43]. Regarding adipsin, the results showing no significant difference between adipsin and severity of DR were not completely in line with the study, which observed that concentrations of adipsin in aqueous humor were higher in DR patients compared to those without DM [40]. The inconsistency may derive from different sample sizes and groups.

In recent years, artificial intelligence and machine learning algorithm have been increasingly applied to detect retinal images for automatic evaluation and classification of DR [44]. In a Chinese multicenter study, the diagnostic performance of a deep learning system model with a neural network for DR presented an AUC of 0.98, with a specificity of 0.96 and a sensitivity of 0.90 [45]. For STDR, Ting et al. developed an algorithm using a deep learning system in a large multiethnic population, with an accuracy of 96%, a sensitivity of 100%, and a specificity of 91.1% [46]. Although machine learning technology is proposed to be a cost-effective and time-saving approach for the identification and grading of DR, with a comparable or even superior sensitivity compared to ophthalmologists and specialists [47], it is difficult to identify early warning indications in the DR development or predict the dynamic progression of DR. A recent systematic review has summarized the prognostic models predicting the development of DR and externally validated their accuracy in a large Dutch type 2 diabetic cohort [48]. It has shown that most of the prediction models, derived from the clinical characteristics, have possibly underestimated the risk of DR. Of note, most of them lack external validation in a separate longitudinal cohort. Compared to the models with the highest C statistics in the review [49, 50], the model in our study presented a comparable accuracy with fewer predicting variables. In comparison with clinical parameters, measurements of adipokines have been increasingly accessible. With the increase in the importance of adipokines and obesity in the clinical and research filed, measurements and application of adipokines in the clinical practice would become more prevalent.

This study has several strengths. First, we developed a prediction model among the patients with mild DR, focusing on the early identification of high-risk population at an early stage and timely intervention to prevent the disease progression to STDR and visual impairment. Second, we utilized a longitudinal cohort for external validation, which the majority of prediction models in predicting DR could not achieve. However, there are several limitations to our study. First, this is a single-center study. Second, due to lack of optical coherence tomography examinations, diabetic macular edema was not included in the STDR group, which may result in a statistical bias. Third, the longitudinal cohort used for validation was relatively small with a relatively short follow-up period. Therefore, a multicenter longitudinal cohort study with a longer duration of follow-up was needed to validate the prediction model.

## 5. Conclusion

In our study, we established a new model, displaying an acceptable performance, to predict the progression to STDR among patients with mild NPDR. The combination of circulating levels of APN, leptin, and FABP4 was capable of identifying high-risk individuals for STDR at an early stage, suggesting that adipokines may play an important role in the pathophysiology of DR development.

## Figures and Tables

**Table 1:** Clinical characteristics of the study participants.

	Case-control set		External cohort
	Training set (*n* = 215)	Testing set (*n* = 91)	Validation set (*n* = 110)
Age (years)	55 (16.00)	53 (17.50)	53 (16.5)
BMI (kg/m^2^)	25.6 (5.35)	25.6 (4.90)	26.3 (4.55)
Duration of diabetes (years)	9.92 (11.92)	9.00 (13.42)	10.0 (12.15)
Hypertension (%)	76 (35.3%)	43 (47.3%)	49 (44.5%)
Gender (male)	114 (53.0%)	48 (52.7%)	51 (46.4%)
Smoke (%)	61 (28.4%)	24 (26.4%)	32 (29.1%)
Laboratory parameters, median (IQR)			
Fasting glucose (mmol/l)	8.16 (4.42)	8.38 (4.78)	9.81 (6.41)
Fasting C-peptide (mIU/l)	1.69 (1.32)	1.40 (1.17)	1.64 (1.16)
2 h C-peptide (mIU/l)	3.61 (3.42)	3.34 (3.76)	3.86 (3.30)
HbA1c (%)	9.40 (2.75)	10.10 (2.85)	9.25 (2.85)
TGs (mmol/l)	1.55 (1.18)	1.65 (1.44)	1.61 (1.08)
TC (mmol/l)	4.83 (1.52)	5.00 (1.87)	4.46 (1.45)
LDL-c (mmol/l)	3.10 (1.21)	3.24 (1.32)	2.90 (1.06)
eGFR (ml/min/1.73m^2^)	117 (38.70)	113 (44.81)	118 (34.32)
UACR			
Normal	134 (62.3%)	52 (57.1%)	77 (70.0%)
Microalbuminuria	58 (27.0%)	26 (28.6%)	24 (21.8%)
Macroalbuminuria	23 (10.7%)	13 (14.3%)	9 (8.2%)
Diabetic retinopathy			
Mild NPDR	140 (65.1%)	60 (65.9%)	110 (100%)
STDR	75 (34.9%)	31 (34.1%)	—

Continuous variables are presented as median (interquartile ranges), and categorical variables are expressed as numbers with percentages. Abbreviations: BMI: body mass index; HbA1c: hemoglobin A1c; TGs: triglycerides; TC: total cholesterol; LDL-c: low-density lipoprotein cholesterol; eGFR: estimated glomerular filtration rate; UACR: urinary albumin-to-creatinine ratio; NPDR: nonproliferative diabetic retinopathy; STDR: sight-threatening diabetic retinopathy.

## Data Availability

Data are currently not available.

## Supplementary Materials

Supplementary 1 Suppl. Figure 1: the flow chart of this study.

Supplementary 2 Suppl. Figure 2: comparisons of clinical parameters between mild NPDR (green color) and STDR group (orange color) in both training and testing sets.

Supplementary 3 Suppl. Figure 3: candidate adipokine selection.
