# Supplementary figures and images for: A Prediction Model for Sight-Threatening Diabetic Retinopathy Based on Plasma Adipokines among Patients with Mild Diabetic Retinopathy
Source: J Diabetes Res. 2023 Oct 25;2023:8831609. doi: 10.1155/2023/8831609 (PMC10620016; doi:10.1155/2023/8831609)

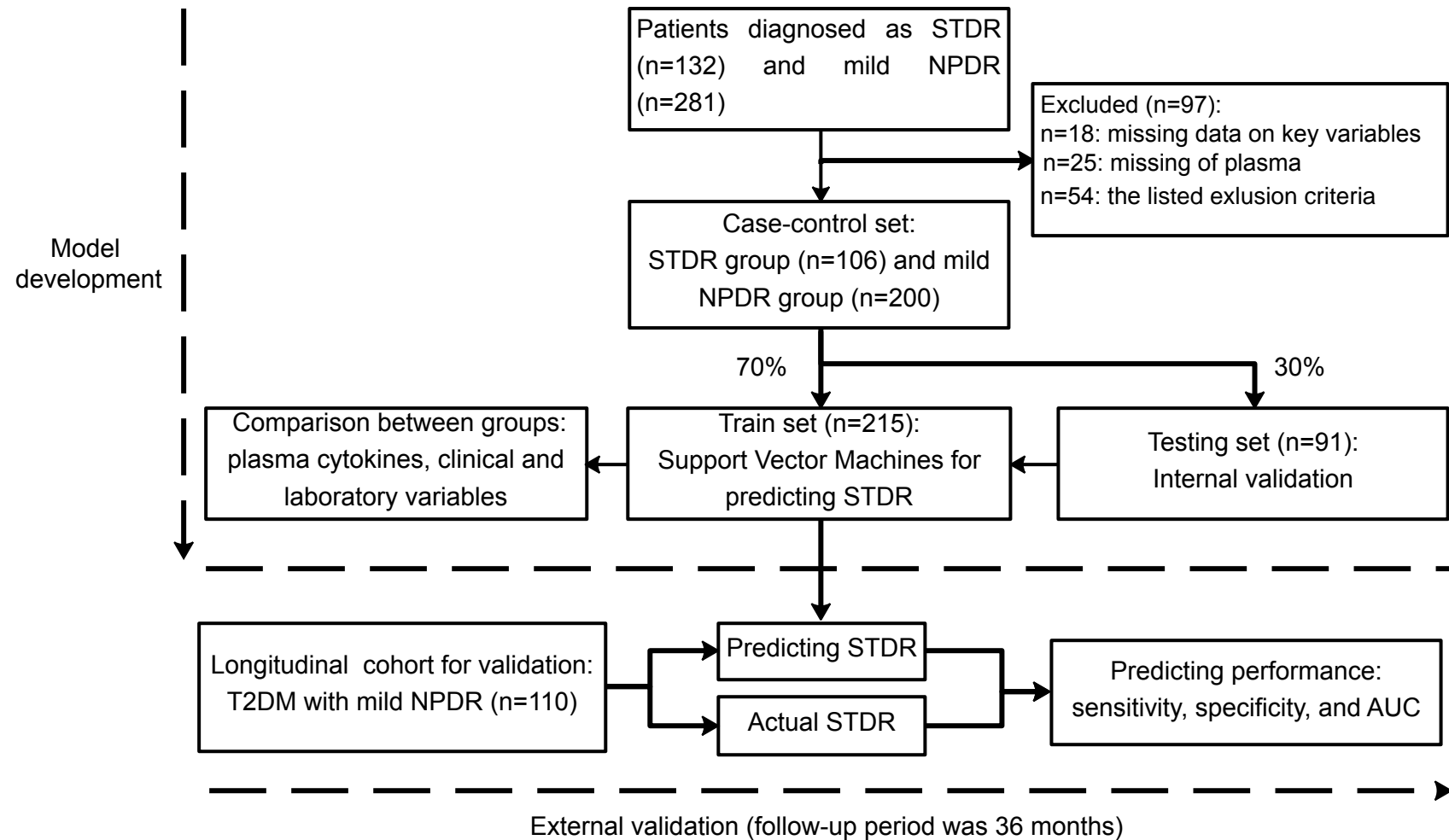

Supplement: Supplementary 1 — Suppl. Figure 1: the flow chart of this study. [file 8831609.f1.pdf]

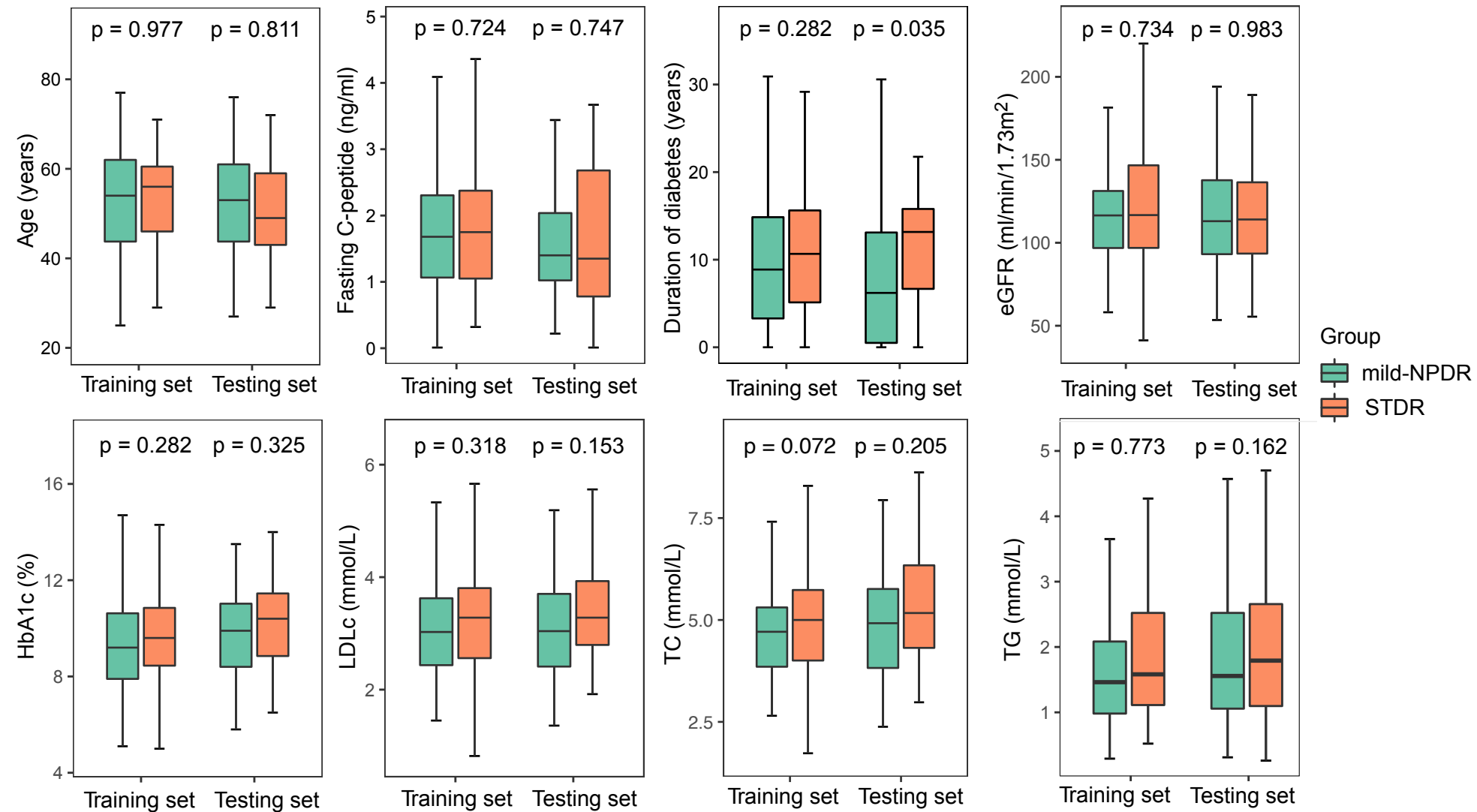

Supplement: Supplementary 2 — Suppl. Figure 2: comparisons of clinical parameters between mild NPDR (green color) and STDR group (orange color) in both training and testing sets. [file 8831609.f2.pdf]

A

PCA – Biplot

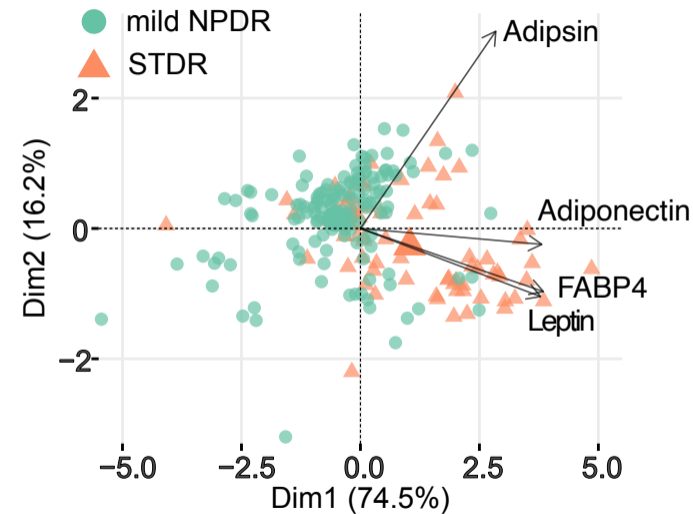

B

Contribution of variables to Dim-1

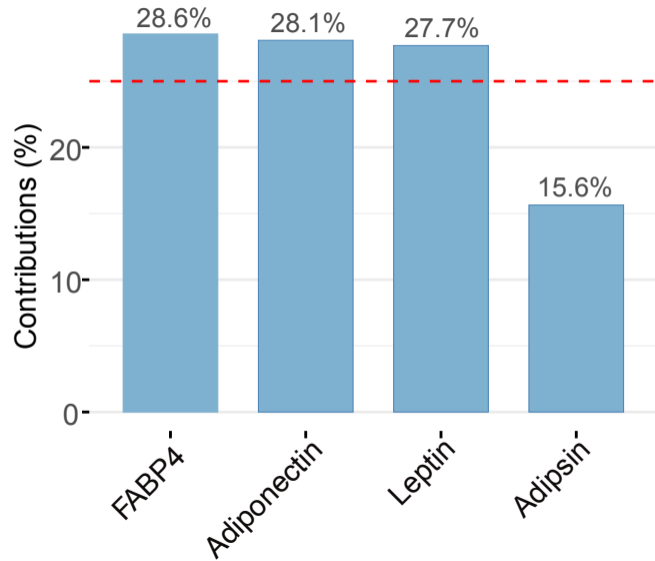

Supplement: Supplementary 3 — Suppl. Figure 3: candidate adipokine selection. [file 8831609.f3.pdf]
